# Broader functionality of language areas at the left middle frontal gyrus in patients with Broca’s area tumors

**DOI:** 10.1016/j.nicl.2025.103860

**Published:** 2025-08-06

**Authors:** Riho Nakajima, Akitoshi Ogawa, Masashi Kinoshita, Takahiro Osada, Hirokazu Okita, Seiki Konishi, Mitsutoshi Nakada

**Affiliations:** a Department of Occupational Therapy, Faculty of Health Science, Institute of Medical, Pharmaceutical and Health Sciences, Kanazawa University, Kanazawa, Japan; b Department of Neurophysiology, Juntendo University School of Medicine, Tokyo, Japan; c Department of Neurosurgery, Faculty of Medicine, Institute of Medical, Pharmaceutical and Health Sciences, Kanazawa University, Kanazawa, Japan; d Department of Physical Medicine and Rehabilitation, Kanazawa University Hospital, Kanazawa, Japan; e Sapiens Life Sciences, Evolution and Medical Research Center, Kanazawa University, Kanazawa, Japan

**Keywords:** Awake mapping, Glioma, Functional connectivity, Centrality, Functional shift

## Abstract

The frontal language area (FLA; left posterior inferior frontal gyrus [pIFG] or Broca’s area), critical for language processing can reorganize in response to lesion progression. While reorganization in the contralateral hemisphere is well known, how reorganization occurs within the ipsilateral hemisphere, especially in the perilesional region, remains unclear. Direct electrical stimulation (DES) during awake surgery enables identification of causal relationships between brain regions and language functions with high spatial resolution. In this study, we investigated cortical reorganization within the ipsilateral hemisphere of the FLA. Seventy-two patients with left hemisphere gliomas were studied. Patients were divided into FLA and non-FLA groups based on whether lesions included the pIFG (n = 10 and n = 62, respectively). All patients underwent DES during a picture-naming task, as recommended by awake surgery guidelines. A subset also underwent resting-state functional MRI (rsfMRI) before surgery to calculate betweenness centrality, an index of network importance of brain areas. DES revealed that the pIFG exhibited positive (impaired) responses to the picture-naming task in both groups. Notably, the frequency of positive responses in the middle frontal gyrus (MFG) was significantly higher in the FLA group than in the non-FLA group. RsfMRI-based network analyses revealed that two areas in the MFG, one in the anterior part and the other in the posterior part, showed higher centrality than surrounding frontal areas in both groups, especially the posterior one. These results suggest that language areas can be observed in the perilesional MFG regions following tumor progression, and raise the possibility that network hubs contribute to maintaining cognitive functions after brain lesions.

## Highlights

• Posterior inferior frontal gurus (pIFG) are known as the frontal language area (FLA).
• We studied the reorganization characteristics of the FLA in brain tumors.
• We performed direct electrical stimulation during surgery and functional MRI at rest.
• Language area extended to the middle frontal gyrus (MFG) when tumors exist on pIFG.
• The MFG, especially its posterior part, was the hub region of the network.

## Graphical abstract

**Figure:**
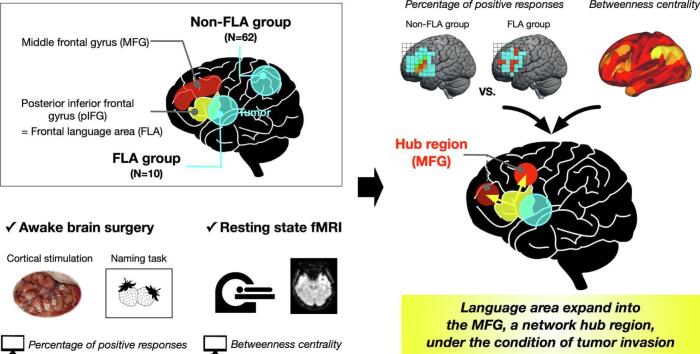

## 1 Introduction

The phenomenon of reallocating brain functions to outside the lesioned area to prevent neurological or cognitive deficits is called functional reorganization (Cargnelutti et al., 2020, Duffau, 2014). Although the frontal language area (FLA; i.e., the left posterior inferior frontal gyrus [pIFG] or Broca’s area) is critical for language functions, cortical damage in the FLA alone does not necessarily cause language deficits (Duffau, 2012, Duffau, 2018), presumably due to functional reorganization. This reorganization can occur in three brain locations: regions in the contralateral hemisphere, ipsilateral regions distant from the lesion, or ipsilateral perilesional regions (Cargnelutti et al., 2020, Nieberlein et al., 2023). Reorganization in the contralateral hemisphere, typically in the same anatomical area on the opposite side of the brain (i.e. right IFG), has often been reported using neuroimaging methods (Cargnelutti et al., 2020, Elkana et al., 2013, Pasquini et al., 2023, Quinones et al., 2023, Saur et al., 2006, Stockert et al., 2020). On the other hand, there are fewer neuroimaging studies for ipsilateral reorganization in regions distant from the lesion, such as the posterior language area, orbitofrontal cortex, and insula (Avramescu-Murphy et al., 2017, Deverdun et al., 2020, Gunal et al., 2018, Stockert et al., 2020). Due to the difficulty in estimating brain activity in perilesional areas covered with neovasculature in the tumor tissue (Hou et al., 2006), reorganization in the peri-lesion has rarely been reported (Gunal et al., 2018, Stockert et al., 2020). Moreover, neuroimaging methods alone cannot determine whether reorganized areas, including perilesional regions, are essential for language functions (Morrison et al., 2016).

The FLA has been implicated in various aspects of language processing, and the most common symptom caused by damage to the area is aphasia (de Oliveira-Souza et al., 2016). Specifically, it is involved in syntactic processing, especially for complex sentences requiring hierarchical structural building (Berwick et al., 2013), and is particularly active when syntactic operations impose greater working memory load (Fiebach and Schubotz, 2006). In addition, the FLA plays a key role in lexical retrieval, verb generation, and verbal fluency during language production (Ardila et al., 2016, Conner et al., 2019, Godefroy et al., 2023, Liégeois et al., 2016). Apart from language-related functions, the FLA is also involved in working memory and executive functions, as well as in action observation and social perception, particularly in relation to the mirror neuron system (Diveica et al., 2023, Keuken et al., 2011, Martin et al., 2025, Mou et al., 2024).

Direct electrical stimulation (DES) during awake surgery for brain tumors provides high-sensitivity, high-spatial-resolution mapping of reorganization, even within perilesional areas (Nakajima et al., 2020, Ng et al., 2023, Nieberlein et al., 2023). This method also enables real-time, causal investigation of the relationship between anatomical structures and specific functions (Yordanova et al., 2019). Previous studies using DES to assess language functions have reported reorganization in the pIFG and its surrounding areas, including the middle frontal gyrus (MFG) (Ng et al., 2023, Saito et al., 2016, Southwell et al., 2016). However, how such reorganization was brought about has not been demonstrated. Given that cortical damage in the FLA does not always result in language deficits (Duffau, 2012, Duffau, 2018), one possible hypothesis would be that perilesional areas in the frontal cortex may compensate by recruiting these regions for language functions typically carried out by the pIFG. We tested this hypothesis by examining whether perilesional areas in patients with pIFG tumors showed positive (impaired) responses to language tasks during DES.

Resting-state functional magnetic resonance imaging (rsfMRI) offers a complementary approach to exploring neural circuits associated with cognitive functions at the whole-brain level (Fox and Raichle, 2007). In network analyses using graph theory, areas that interact with many other regions, commonly referred to as “hubs,” are considered critical to brain networks (Bullmore and Sporns, 2009, Crossley et al., 2014). Betweenness centrality (BC) is an index of network importance based on functional connectivity, indicating regions critical to information flow within a brain network (Freeman, 1977, Zuo et al., 2012). We have previously reported that regions with high BC play central roles in brain networks, with BC levels correlating with behavioral performance (Fujimoto et al., 2020). Combining rsfMRI network analysis with DES during awake surgery can thus provide valuable insights into brain functions (Nakajima et al., 2024, Yordanova et al., 2019).

This study aimed to characterize functional reorganization in the ipsilateral hemisphere after tumor invasion to the pIFG using DES during awake surgery and preoperative rsfMRI. Based on the hypothesis that perilesional areas may be recruited to support language function, we examined whether these perilesional areas, as well as the FLA itself, exhibited positive responses to DES. We divided the patients into two groups, FLA and non-FLA, depending on whether the preoperative tumor extended to the pIFG. During surgery, patients performed a picture-naming task with DES, a standard method for evaluating the function of the language area, including the FLA, as recommended by guidelines (De Witte and Marien, 2013, Herbet, 2021, Kayama and Guidelines committee of the Japan awake surgery conference, 2012, Young et al., 2021). This method enables us to identify positive (impaired) and normal (unimpaired) responses on the cortical surface. We also conducted rsfMRI before surgery with a subset of patients and performed network analyses to examine the characteristics of the areas where cortical reorganization occurred in patients with pIFG tumor invasion.

## 2 Materials and methods

### 2.1 Participants

A total of 184 patients with World Health Organization (WHO) grades 2, 3, and 4 gliomas, categorized based on the 2021 WHO Classification of Tumors of the Central Nervous System, who underwent awake surgery for glioma resection at Kanazawa University Hospital between May 2014 and November 2023 were enrolled. Patients were excluded based on the following criteria: presence of tumors on the right side (n = 90), unsuccessful awake monitoring or recording (n = 11), and lack of performance in a picture-naming task during awake surgery (n = 11) (Fig. S1). Consequently, 72 patients were included in this study (mean age ± standard deviation: 47.5 ± 13.5 years). Handedness was right in 69 patients and left in 3 patients. Given the relevance of handedness for language lateralization, we confirmed hemispheric language dominance in all left-handed patients using preoperative functional MRI with a picture-naming task, which demonstrated left-hemisphere activation in each case. We defined the FLA as the common definition of Broca’s area, synonymous with the pars opercularis and pars triangularis in the pIFG (Brodmann’s areas 44 and 45) (Tremblay and Dick, 2016). To assess language function before surgery, all patients completed a picture-naming task with high-frequency words from the Supplementary Test of the Standard Language Test of Aphasia (SLTA-ST) (Brain function test committee, 1999).

Patients were divided into two groups based on tumor location (FLA group: n = 10; non-FLA groups: n = 62; see Fig. S2). The FLA group included patients whose tumors overlapped with the pIFG, whereas the non-FLA group included those with tumors located outside the pIFG, primarily in the frontal, parietal, or temporal lobes. Tumor location was determined based on fluid-attenuated inversion recovery (FLAIR) hyperintensities for grades 2 and 3 gliomas and gadolinium enhancements in T1-weighted images for grade 4 gliomas. Demographic and clinical characteristics of each patient group are summarized in Table 1. Written informed consent was obtained from all patients. This study was conducted in accordance with the guidelines of the Internal Review Board and approved by the Medical Ethics Committee of Kanazawa University (No. 1797 and 3322).

**Table 1:** Demographic and clinical characteristics of participants.

Characteristics	Value		
	Non-FLA group (n = 62)	FLA group (n = 10)	P-value
Age			0.43
Mean ± SD	48.0 ± 14.2	44.4 ± 7.1	
Range	16–75	34–55	

Sex			0.94
Male	38	6	
Female	24	4	

WHO grade			0.42
2	25	3	
3	13	4	
4	24	3	

Preoperative tumor volume (cm^3^)			0.81
Mean ± SD	31.9 ± 34.8	34.7 ± 32.1	

Preoperative picture-naming score (%)			0.83
Mean ± SD	99.0 ± 2.5	99.2 ± 1.6	

Amplitude of direct electrical stimulation (mA)			0.73
Mean ± SD	4.1 ± 1.2	4.0 ± 1.0	
Range	2.5–6.0	3.0–6	

### 2.2 MRI-based group classification

**Fig. 1:**
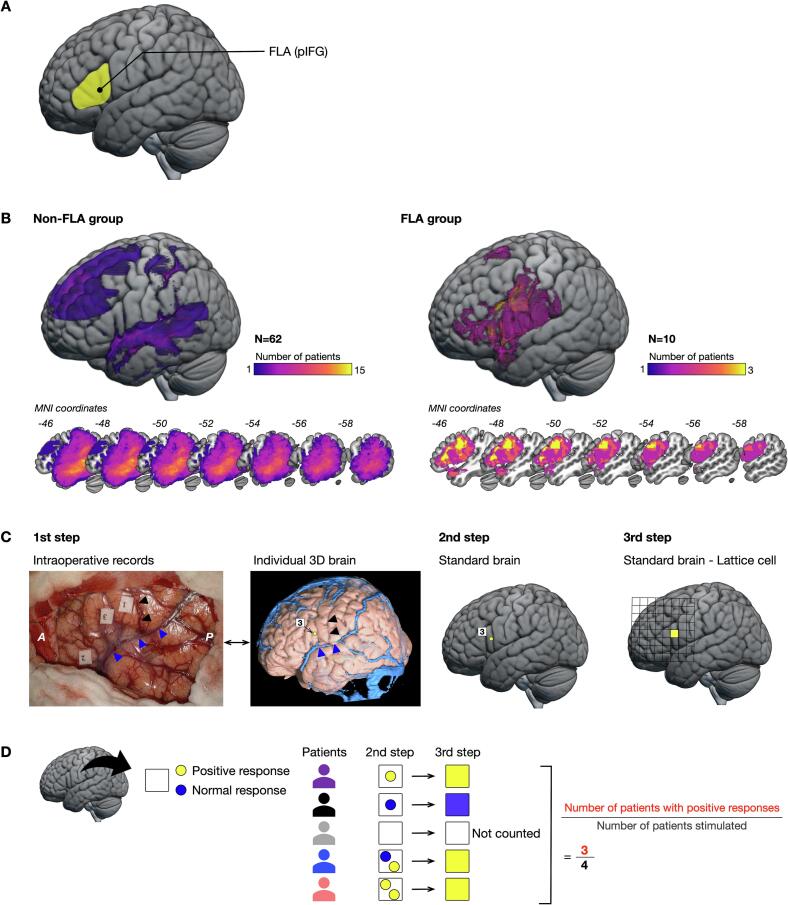
Methods of this study. (A) The location of the frontal language area (FLA) or posterior inferior frontal gyrus (pIFG) in the left hemisphere, which corresponds to the opercular and triangular parts of the frontal lobe. (B) Maps of tumor overlap in the non-FLA and FLA groups. Yellow regions show the highest overlap in each group. (C) The schema for identifying positive points. Step 1: Positive points were identified using number tags in the intraoperative video records. The locations of these points were determined based on spatial relationships to anatomical landmarks, such as vessel, gyri, and sulci, as well as the edge of the resection cavity. Blue triangle, Sylvian fissure; black triangle, central sulcus; P, posterior; A, anterior. Step 2: All points were transferred to the standard brain. Step 3: Positions of positive points were mapped in lattice cells for statistical analysis. (D) If at least one positive point is found within a square in the second step, the corresponding square is classified as positive. Multiple positive points within a square are counted as a single positive square. (For interpretation of the references to colour in this figure legend, the reader is referred to the web version of this article.)

All patients underwent preoperative structural MRI, including T1-weighted and FLAIR images, as part of standard care at Kanazawa University Hospital (T1-weighted images: repetition time [TR], 6.04 ms; echo time [TE], 2.4 ms; voxel size, 0.94 × 0.94 × 1.00 mm; field of view [FOV], 240 × 240 × 180 mm; FLAIR images: TR, 6000 ms; TE, 114.5 ms; voxel size, 0.70 × 0.47 × 0.47 mm; FOV, 180 × 240 × 240 mm). MRI was performed using a 3.0-T MR scanner (Signa Excite HDx 3.0 T [GE Healthcare, Little Chalfont, UK] or Philips Ingenia [Philips Healthcare, Best, the Netherlands]). During the preprocessing of structural MRI, the T1-weighted and FLAIR images were spatially normalized to the Montreal Neurological Institute (MNI) template using unified models implemented in SPM12 (https://www.fil.ion.ucl.ac.uk/spm/). This method enables normalization of images to the MNI space by performing tissue matching based on the probability maps for gray matter, white matter, and cerebrospinal fluid, even in the presence of lesions (Ashburner and Friston, 2005). The structural images were resliced to a 1.0 mm isovoxel resolution. Preoperative tumor areas were manually segmented using MRIcron software (people.cas.sc.edu/rorden/mricron/) (Brett et al., 2001) for each patient. Each segmentation was performed by the first author (R.N.) and validated by a neurosurgeon (M.K.). Patients in the FLA group (n = 10) were defined as having segmented tumors overlapping with Brodmann’s areas 44 or 45 by at least 50 voxels (Biesbroek et al., 2013) (Fig. 1A). Maximum tumor overlap across patients was observed in the deep part of the middle temporal gyrus in the non-FLA group and the deep part of the posterior premotor area in the FLA group (Fig. 1B).

### 2.3 Spatial topography of cortical stimulations

Awake mapping was performed during all surgeries to preserve language function, following the established awake surgery guidelines (Kayama and Guidelines Committee of the Japan awake surgery, 2012). Each patient underwent surgery using the asleep-awake-asleep technique (Duffau et al., 2002). When patients were awake, cortical regions were stimulated using a bipolar electrode with 5-mm-spaced tips delivering a biphasic current (pulse frequency of 60 Hz, pulse duration of 0.2 ms, amplitude between 1.5 and 6 mA; Neuromaster, Nihon Kohden Co., Tokyo, Japan). To determine DES thresholds, stimulation intensity was incrementally increased from 1.5 mA until anarthria or motor responses were observed at the primary motor area. This determined intensity was then used for subsequent DES (Kayama and Guidelines Committee of the Japan awake surgery, 2012). Language function was assessed using a picture-naming task with high-frequency words based on SLTA-ST (Brain-function-test-committee, 1999), as described previously (Nakajima et al., 2024). The high-frequency words in the SLTA-ST were selected from “Electric data of research on basic vocabulary for learners of Japanese” (https://mmsrv.ninjal.ac.jp/bvjsl84/en/index.html) and list of Corpora (https://clrd.ninjal.ac.jp/en/index.html) by the National Institute for Japanese Language and Linguistics (Brain function test committee, 1999). Patients were asked to name pictures presented on a monitor using PowerPoint® (Microsoft, Redmond, WA). Incorrect responses were classified as “positive responses”, which reflect regions essential for language function (Tate et al., 2014), and correct responses as “normal responses.” The positive responses included anomia, phonemic paraphasia, semantic paraphasia, speech arrest, and anarthria (Leonard et al., 2019, Sarubbo et al., 2020). Based on established guidelines of awake surgery (Kayama and Guidelines Committee of the Japan awake surgery, 2012), each brain site was stimulated up to three times; sites with positive responses in two trials were labeled positive points, while others were labeled normal points. The total number of stimulated sites varied by patient based on tumor location and surgical planning. Positive points were preserved for resection to prevent postoperative aphasia. Response classifications were performed by a speech therapist (H.O.) or an occupational therapist (R.N.), later reviewed by a senior neurosurgeon, who was blinded to the initial classifications, using intraoperative video recording.

In the frontal lobe, DES primarily induces five types of language-related errors: anomia, phonemic paraphasia, semantic paraphasia, speech arrest, and anarthria. Symptoms of paraphasia and anarthria/speech arrest should be distinguished as they arise from different mechanisms and have distinct clinical implication. Aphasia, defined as the loss or impairment of verbal communication, is classified based on fluency, comprehension, and picture-naming ability (Sinanović et al., 2011). In contrast, anarthria, specifically related to vocal system dysfunction induced by DES, affects articulatory control without impairing linguistic processing (Lucchelli and Papagno, 2005). Additionally, speech arrest is a distinct phenomenon caused by the inhibition of muscular activity around the mouth or the disruption of speech production coordination. These effects can result from DES applied to the primary motor area or the negative motor network, respectively (Lu et al., 2021, Rech et al., 2019, Tomasino et al., 2024). The negative motor network refers to specific cortical regions in the frontal lobe where DES elicits an arrest of voluntary movements, including the lateral frontal area just rostral to the primary face motor area and the medial frontal area just rostral to the supplementary motor area (Enatsu et al., 2013). Accordingly, only aphasia-related naming errors, such as anomia, phonemic paraphasia, and semantic paraphasia, were included in the subsequent analyses, in line with previous studies (Bährend et al., 2021, Saito et al., 2020).

Positive and normal points were identified from intraoperative video recordings. Numbered tags marked the locations of positive points, while normal points were determined using the video-recorded bipolar electrode tip. These anatomical locations were analyzed through the following three steps (Fig. 1C). First, positive and normal points were plotted on individual T1-weighted images using iPlan Stereotaxy 3.0 software (BrainLab, München, Germany). Locations were determined with reference to anatomical landmarks (i.e., gyri, sulci, and vessels) and the edge of the resection cavity (Tate et al., 2014). Second, positive and normal points were mapped to the MNI coordinates using MRIcroGL software (github.com/rordenlab/MRIcroGL/). Third, locations were converted into square sections on a lattice (10 × 10 mm) covering the FLA (Nakajima et al., 2024, Southwell et al., 2016) (Fig. 1C). Squares containing at least one positive point were classified as positive (Fig. 1D). The stimulated locations on the MNI template were cross-validated against individual T1-weighted images by the first author (R.N.) and validated by two neurosurgeons (M.K. and M.N.).

### 2.4 rsfMRI

In total, 44 patients, including 37 patients in the non-FLA group (mean age: 49.2 ± 14.3 years; 22 males, 15 females) and seven in the FLA group (mean age: 42.1 ± 6.8 years; 5 males, 2 females), underwent rsfMRI before surgery (Fig. S2). Patient details are shown in Table 2. As an age-matched control, we recruited 38 patients with right cerebral hemispheric gliomas (mean age: 46.9 ± 15.7 years; 24 males, 14 females; WHO grade 2, n = 19; WHO grade 3, n = 12, WHO grade 4, n = 7). The rsfMRI was acquired using single-band or multi-band gradient-echo echo-planar imaging (EPI) sequences (for single-band EPI, TR = 2.5 s, TE = 30 ms, voxel size = 3.3 × 3.3 × 3.2 mm, matrix size = 64 × 64, 40 slices with 0.8 mm gap, number of volumes = 240; for multi-band EPI, TR = 1.5 s, TE = 30 ms, voxel size = 2.4 × 2.4 × 2.4 mm, matrix size = 96 × 96, 54 contiguous slices, multi-band factor = 3, number of volumes = 300) in a 3.0 Tesla MR imaging GE scanner (Signa™ Excite HDx 3.0 T; GE Healthcare, Little Chalfont, UK) or Philips scanner (Philips Ingenia; Philips Healthcare). During the rsfMRI scan, the patients were asked to keep their eyes open.

**Table 2:** Demographic and clinical characteristics of participants who underwent rsfMRI.

Characteristics	Value		
	Non-FLA group (n = 37)	FLA group (n = 7)	P-value
Age			0.21
Mean ± SD	49.2 ± 14.3	42.1 ± 6.8	
Range	19–75	34–49	

Sex			0.55
Male	22	5	
Female	15	2	

WHO grade			0.089
2	14	3	
3	9	4	
4	14	0	

Preoperative tumor volume (cm^3^)			0.54
Mean ± SD	31.0 ± 32.0	39.4 ± 35.5	

Amplitude of direct electrical stimulation (mA)			0.57
Mean ± SD	3.9 ± 1.2	3.6 ± 0.56	
Range	2.5–6.0	3.0–4.5	

Preprocessing of rsfMRI images was conducted using SPM12, FSL (fsl.fmrib.ox.ac.uk/fsl/fslwiki/), and the Human Connectome Project (HCP) pipelines (Glasser et al., 2016, Glasser et al., 2013) similar to previously reported procedures (Nakajima et al., 2022, Osada et al., 2024, Osada et al., 2021, Rison et al., 2024). Preprocessing included slice timing-correction, realignment, spatial normalization to the MNI template with a resolution of 2.0 mm isovoxel, ICA-FIX denoising (Salimi-Khorshidi et al., 2014), and projection onto the 32 k fs_LR surface space using MSMSulc (Glasser et al., 2016, Robinson et al., 2014) and Ciftify (Dickie et al., 2019). Then, the global signal was regressed from the time-series data, followed by spatial smoothing (full width at half maximum = 6 mm).

The preprocessed rsfMRI data were averaged across vertices in each of the 360 cerebrocortical parcels (i.e., functional areas) provided by the HCP (Glasser et al., 2016). Temporal correlation coefficients between parcels were calculated and transformed to Fisher’s z-values as functional connectivity. The top 10 % of connections were thresholded to create a binary undirected network of the resting-state cerebral network. We employed BC, which measures the proportion of the shortest paths between all parcel pairs in the cerebral network (Brandes, 2001, Freeman, 1978, Fujimoto et al., 2020) to assess network importance. The Brain Connectivity Toolbox (Rubinov and Sporns, 2010) was used to define the cerebral network and calculate the BC of each parcel. The BC of parcel *i* (*b_i_*) was calculated as follows:

(1) $$b_{i}=\frac{1}{\left(N-1\right)\left(N-2\right)}\sum_{h\neq i,h\neq j,j\neq i}\frac{\rho_{\mathrm{hj}}\left(i\right)}{\rho_{\mathrm{hj}}}$$

where *ρ_hj_*(*i*) is the number of shortest paths between parcels *h* and *j* that pass through parcel *i*, *ρ_hj_* is the number of shortest paths between parcels *h* and j, and *N* is the number of parcels. If information is conveyed in the cerebral network along the shortest path, a parcel with high BC mediates a high proportion of information traffic. Such parcels may control the information passage or act as bottlenecks for information flow.

### 2.5 Statistical analysis

To compare the demographic and clinical characteristics of participants between groups (FLA vs. non-FLA), t-tests and chi-square tests were performed. The FLA and its surrounding areas were divided into square sections, and the ratio (%) of the number of patients with positive responses relative to the total number of patients stimulated in each square section was calculated. A chi-square test was used to compare these ratios between the FLA and non-FLA groups.

To analyze the rsfMRI results, we first tested the normality of each area (n = 14) in both the FLA and non-FLA groups using Kolmogorov-Smirnov test. The results indicated that the data were generally normally distributed across areas in both groups (Kolmogorov–Smirnov test, p > 0.05). In the non-FLA group, however, p-values were close to the threshold in areas 44, a9-46v, and 8C (p = 0.044, p = 0.046, and p = 0.049, respectively). Given the multiple comparisons and the marginal nature of these p-values, the assumption of normality was considered generally satisfied. Sphericity was tested using Mauchly’s test prior to performing an analysis of variance (ANOVA) for the initial three-way ANOVA, which included more than two levels for the within-subject factor. As the sphericity was violated (p < 0.05), the p-values were corrected using Huynh-Feldt methods. Statistical comparisons of BC values were performed using a three-way ANOVA, with parcel or parcel group as a within-subject factor, and patient group (FLA vs. non-FLA) and MRI scanner as between-subject factors. The MRI scanners were treated as a controlling factor. The p-values for the F statistic were adjusted using Huynh-Feldt correction. For subsequent analyses using three-way ANOVAs (2 × 2 × 2 design), in which each factor had only two levels, the assumption of sphericity was not applicable and therefore not tested. We did not perform exploratory post-hoc testing across all parcels. Rather, based on the significant main effect of area (see Fig. 4C), we conducted hypothesis-driven comparisons within the middle frontal gyrus (MFG) to test whether BC was particularly elevated in specific subregions (8C and a9-46v), as suggested by the DES results. For the age-matched control group, BC values were analyzed using a two-way ANOVA, with parcel group as a within-subject factor and MRI scanner as a between-subject factor, which was also treated as a controlling factor. All statistical analyses were performed using JMP Pro version 16.2.0 (SAS Institute Japan Inc., Tokyo, Japan) and SPSS Statistics version 29.0.1.0 (SPSS Inc., Chicago, IL., USA).

## 3 Results

### 3.1 Language assessment before the surgery

The naming-task scores before surgery were 99.0 % in the non-FLA group and 99.2 % in the FLA group, showing no significant difference between groups (p = 0.83) (Table 1). Two patients exhibited mild aphasia, which was determined based on the number and types of errors observed during a preoperative naming-task. Both patients made a few semantic errors; however, these did not interfere with language assessments during awake surgery.

### 3.2 Intraoperative findings

**Fig. 2:**
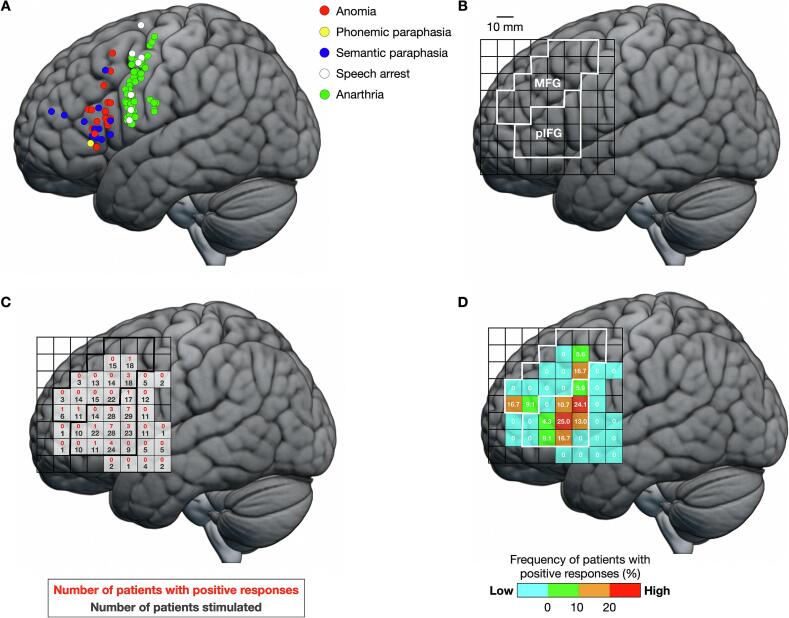
Positive responses for all patients. (A) Distribution of the positive points. Each region was stimulated up to three times. Regions where multiple types of errors were observed in at least two trials were classified as having positive responses. Red, anomia; yellow, phonemic paraphasia; blue, semantic paraphasia; white, speech arrest; green, anarthria. (B) The defined regions for the posterior inferior frontal gyrus (pIFG) and middle frontal gyrus (MFG). (C) Raw data of mapping points, with the number of patients stimulated (lower stand, black letter) and number of patients with positive responses per square (upper stand, red letter). (D) The frequency of positive responses was calculated as the ratio (%) of patients with positive responses to total patients stimulated. Areas with high frequencies of positive responses are indicated by warm colors and areas with low positive responses are indicated by cool colors. One side of a square represents 10 mm. (For interpretation of the references to colour in this figure legend, the reader is referred to the web version of this article.)

In total, 44 patients received DES in and around the pIFG. Notably, two patients who had mild aphasia before surgery did not receive stimulation in or around the pIFG. Overall, 96 positive and 588 normal responses were observed during DES in the picture-naming task. Positive responses included anarthria (58 points), anomia (18 points), phonemic paraphasia (1 point), semantic paraphasia (12 points), and speech arrest (7 points) (Fig. 2A). The MNI coordinates of the positive points are summarized in Table S1. We then focused on 31 stimulation points associated with aphasia-related symptoms, including anomia, phonemic paraphasia, and semantic paraphasia. The percentage of patients with positive responses among the stimulated patients was calculated for each square in the pIFG and surrounding areas including the MFG (Fig. 2B, C, and D). While positive points were frequently observed in the pIFG, they were also found in the MFG at a low frequency.

**Fig. 3:**
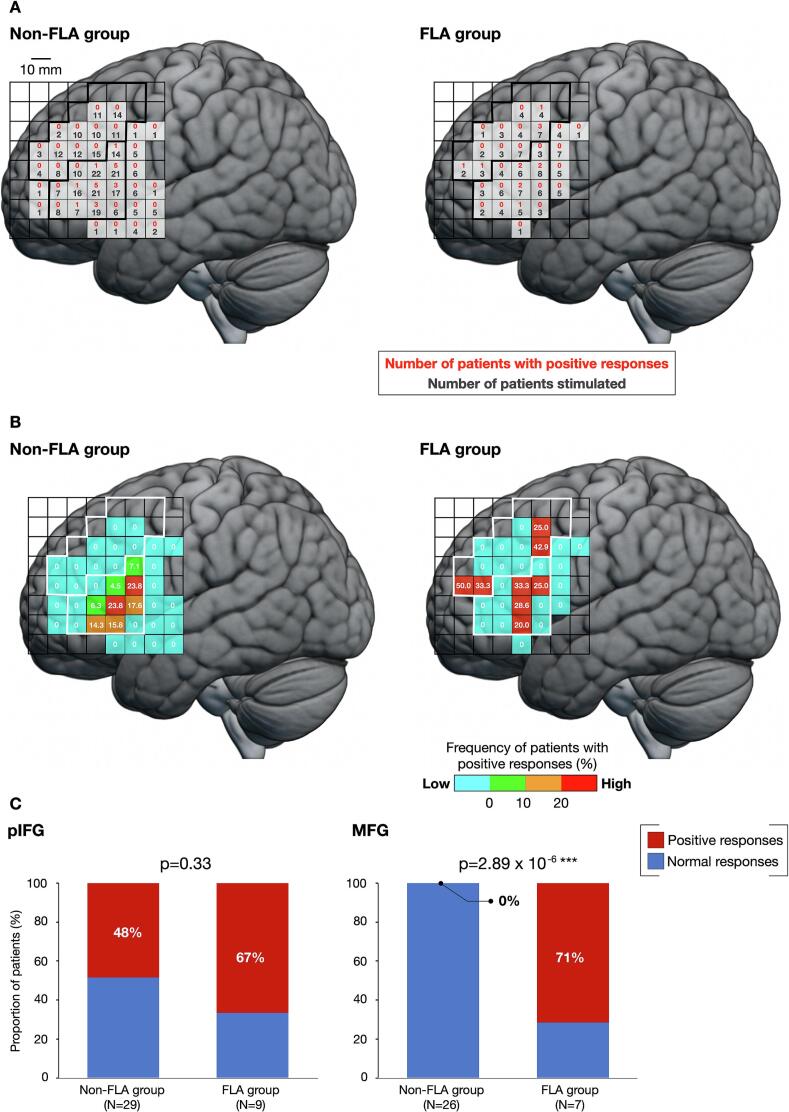
Intraoperative findings in two groups. (A) Raw data of mapping points in the non-FLA (left) and FLA groups (right), with the number of patients stimulated (lower stand, black letter) and number of patients with positive responses per square (upper stand, red letter). (B) The frequency of positive responses for the non-FLA and FLA groups. Warm and cool colors indicate areas with high and low frequencies of positive responses, respectively. One side of a square represents 10 mm. (C) Comparison of the frequencies of positive response between the non-FLA and FLA groups. Red, positive responses; blue, normal responses. ^***^P < 0.001. FLA, frontal language area. (For interpretation of the references to colour in this figure legend, the reader is referred to the web version of this article.)

Next, we divided the patients into FLA and non-FLA groups according to the tumor location (n = 10 and n = 62, respectively) and compared the distribution of positive points between the groups. In both groups, positive points were consistently observed in the pIFG (Fig. 3A and B). Although only normal points were detected outside the pIFG in the non-FLA group, positive points were also observed in the MFG in the FLA group (6 points from 5 patients) (Fig. 3A and B). Notably, positive points in the FLA group clustered in two separate foci in the anterior and posterior parts of the MFG. Analysis of the proportion of positive responses revealed no significant differences in the pIFG between groups (*χ^2^*(1) = 0.93, *P* = 0.33, chi-squared test) (Fig. 3C, left). However, the proportion of positive responses in the MFG was significantly higher in the FLA group than in the non-FLA group (*χ^2^*(1) = 21.9, *P* = 2.89 × 10^−6^, chi-squared test) (Fig. 3C, right).

**Fig. 4:**
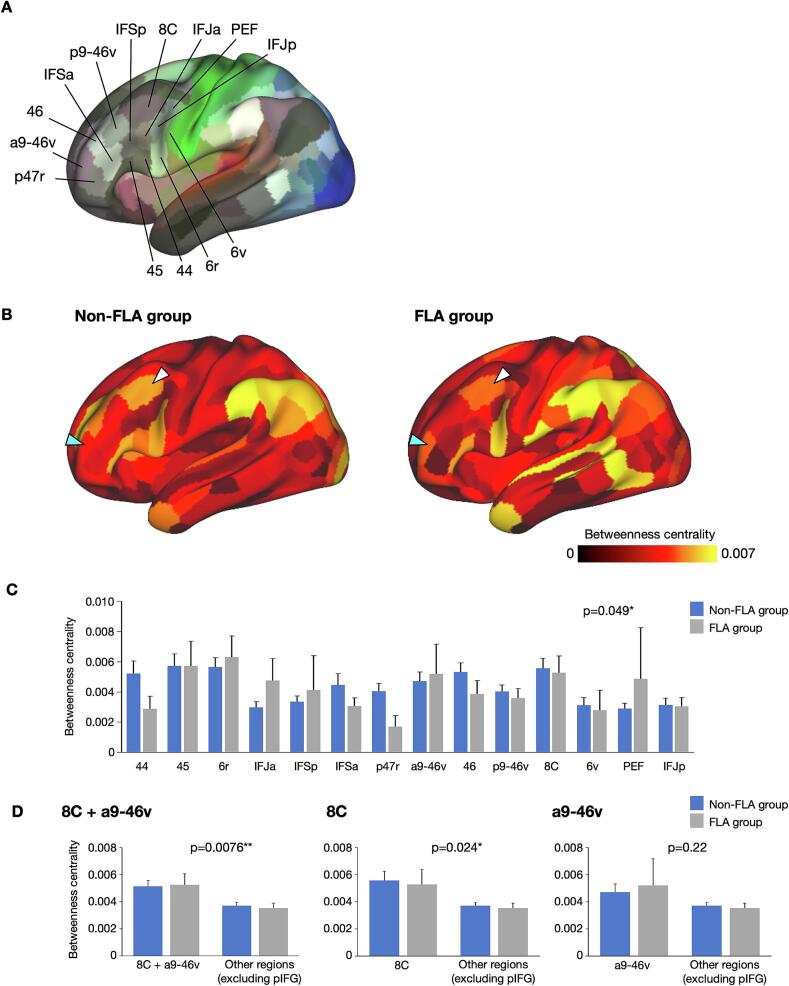
Results of rsfMRI data analysis. (A) Analyzed parcels in the posterior inferior frontal gyrus (pIFG) and surrounding areas. (B) Betweenness centrality (BC) in the non-FLA and FLA groups. The middle frontal gyrus (MFG) was divided into two parts: anterior part (a9-46v, cyan triangles) and the posterior part (8C, white triangles). (C) BC values for the non-FLA (blue) and FLA (gray) groups across each parcel area and patient group. (D) BC values for the MFG (8C + a9-46v) and other surrounding regions excluding the pIFG in the non-FLA (blue) and FLA (gray) groups. Results of a three-way ANOVA with parcel areas (8C + a9-46v vs. other regions, 8C vs. other regions, and a9-46v vs. other regions) as a within-subject factor, and patient groups and MRI scanner (included to control for potential scanner-related effects) as between-subject factors. Error bars indicate the standard error of means. *P < 0.05, ^**^P < 0.01. ANOVA, analysis of variance; FLA, frontal language area. (For interpretation of the references to colour in this figure legend, the reader is referred to the web version of this article.)

In the non-FLA group, tumors extended to the MFG in 16 patients, whereas the remaining 46 patients did not have lesions in the MFG (Fig. S3A). Excluding patients with MFG tumors from the non-FLA group did not alter the trend in the percentage of positive responses (40 % in the pIFG and 0 % in the MFG) (Figs. S3B and S3C). Further analysis in the FLA group was not performed due to the small number of cases. Additionally, the same analysis was performed separately for WHO grades 2 and 3 tumors and grade 4 tumors. The results were similar to the overall results, showing that the proportion of positive responses on the MFG remained significantly higher in the FLA group than in the non-FLA group across both grades (Fig. S4).

### 3.3 Betweenness centrality in rsfMRI connectivity

We analyzed rsfMRI connectivity and calculated the BC of the pIFG and its surrounding regions based on parcels (i.e., functional areas) (Fig. 4A). Areas with high BC were considered functional hubs in the whole-brain network. Among the analyzed parcels in the frontal lobe, areas 6r, 44, and 45 in the pIFG showed relatively high BC (Fig. 4B). In addition, areas a9-46v and 8C in the anterior and posterior parts of the MFG, respectively, also showed high BC. These areas corresponded to the areas with positive responses in the FLA group (Fig. 3B and Fig. S5).

A three-way ANOVA, with parcel areas as a within-subject factor and both patient group (non-FLA/FLA) and MRI scanner (included to control for potential scanner-related effects) as between-subject factors, revealed a significant main effect of area (F(13, 533) = 1.88, *P* = 0.049, after Huynh-Feldt correction, *ε* = 0.74), indicating that BC values varied across different frontal parcels (Fig. 4C). No significant main effects of patient group (F(1, 41) = 0.36, *P* = 0.55) or MRI scanner (F(1, 41) = 3.05, *P* = 0.088), nor interaction effects (area × group: F(13, 533) = 0.86, *P* = 0.57; area × scanner: F(13, 533) = 1.03, *P* = 0.41, all after Huynh-Feldt correction, *ε* = 0.74), were observed. Given the significant main effect of area and our interest in the MFG, based on the results of DES, we focused our further analysis on the MFG. We conducted an additional ANOVA with the parcel groups (MFG [8C + a9-46v, 8C, a9-46v] vs. other regions) as a within-subject factor and patient group and MRI scanner (included to control for potential scanner-related effects) as between-subject factors. BC in the MFG (8C + a9-46v) was significantly higher than that in other frontal regions excluding the pIFG (areas 6r, 44, and 45) (F(1, 41) = 7.88, *P* = 0.0076), while there were no significant main effects of patient group (F(1, 41) = 0.036, *P* = 0.85) or scanner (F(1, 41) = 1.07, *P* = 0.31), and no significant interaction effects (area × group: F(1, 41) = 0.16, *P* = 0.70; area × scanner: F(1, 41) = 0.87, *P* = 0.36) (Fig. 4D, left). Within the MFG, we further explored whether the higher BC was driven by specific subregions. This analysis revealed that BC in the posterior MFG (8C) was significantly higher than that in other regions (F(1, 41) = 5.48, *P* = 0.024), whereas BC in the anterior MFG (a9-46v) did not show significant differences (F(1, 41) = 1.54, *P* = 0.22) (Fig. 4D, middle and right). No significant main effects or interactions were found for patient group or scanner in either analysis.

To confirm the robustness of BC findings, we also analyzed binary undirected networks constructed using different thresholds (top 7.5 %, 12.5 %, and 15.0 %) for resting-state functional connectivity (Fig. S6), in addition to the originally used top 10 % threshold. This approach, commonly adopted in graph-theoretical analyses, helps ensure that the results are not dependent on a specific threshold. Across all threshold conditions, the MFG (8C + a9-46v) consistently exhibited higher BC than other areas (7.5 %: F(1, 41) = 9.96, *P* = 0.0030; 12.5 %: F(1, 41) = 9.20, *P* = 0.0042; 15.0 %: F(1, 41) = 5.58, *P* = 0.023), with no significant main effects or interaction for patient group or scanner. Additionally, BC in the left hemisphere was calculated using an age-matched control dataset of patients with tumors in the right hemisphere (Fig. S7A). The results showed similar trends to those in the non-FLA and FLA groups (Fig. S7B). Specifically, the MFG (8C + a9-46v) and posterior MFG (8C) demonstrated significantly higher than other regions (8C + a9-46v, F(1, 36) = 5.05, *P* = 0.031; 8C, F(1, 36) = 5.07, *P* = 0.031), while the anterior MFG (a9-46v) did not show a significant difference (F(1, 36) = 1.17, *P* = 0.29).

## 4 Discussion

In the current study, we revealed the characteristics of changes in the ipsilateral hemisphere of the FLA group by DES during the performance of a language task and functional connectivity during rest. In the awake DES mapping, most positive points were found in the pIFG in both groups, whereas only the FLA group showed positive points in the MFG. Network analyses revealed that the MFG clusters identified by DES corresponded to high-centrality areas, i.e., network hub regions. These results suggest that language areas were observed in the perilesional areas in the MFG, and that functional involvement of network hub regions might represent a compensatory process.

Positive points in the FLA group were distributed in the pIFG and MFG, but those of the non-FLA group were limited to the pIFG. The positive points located mainly in the pIFG of the FLA and non-FLA groups may correspond to the classical language area. Previous DES studies demonstrated that the distribution pattern of positive points could change when tumors invade the language area (Nakajima et al., 2024, Saito et al., 2016). Therefore, relatively wider distribution of language areas, including the MFG, observed in figures of prior DES studies, may reflect functional reorganization due to tumor invasion to the pIFG (Lu et al., 2021, Ng et al., 2023, Saito et al., 2016). A limited number of previous studies have reported the involvement of the MFG in language functions, such as syntactic processing (Li et al., 2024, Yu et al., 2022). Moreover, the left MFG plays a critical role in second-language learning, including speaking and word reading (Sander et al., 2023, Yokoyama et al., 2013). These findings may support the idea that the MFG has the potential to compensate for the original language areas when they are damaged.

Recently, the usefulness of rsfMRI for identifying language areas before surgery has attracted attention. Previous study has reported a high degree of agreement between the language related regions identified using rsfMRI—such as the IFG, angular gyrus, superior and middle temporal gyri—and those mapped by the DES during awake surgery (Lemée et al., 2019). On the other hand, hubs identified using graph-theoretic analysis are not necessarily specific to language function. Furthermore, the relationship between the network hub regions and positive locations identified in awake mapping remains elusive. At the individual level, a previous report revealed that surgical resection of an area of high functional centrality can cause postoperative language deficits (Lee et al., 2021). Because the location of hubs may be changed by tumor effects (Tuovinen et al., 2016), individual-level analyses may reveal associations between awake mapping results and functional connectivity hubs.

The spatial extent of DES data was limited to the surgical area, as these data were obtained for surgical purposes rather than primarily for research. Ideally, to study functional reorganization, it would be more appropriate to compare language areas of patients with gliomas to those of healthy individuals or to compare language areas before and after the appearance of lesions in the same individual. Therefore, this study compared the distribution of language areas between the FLA and non-FLA groups, an approach in line with previous studies (Nakajima et al., 2024, Saito et al., 2016).

Another limitation is the potential sampling bias of stimulation points between the FLA and non-FLA groups. In particular, the number of cases in the FLA group was relatively small because patients with preoperative language deficits due to lesions in the FLA were inapplicable for awake surgery and were excluded from this study. Similarly, the number of patients who underwent rsfMRI was also limited due to the clinical prioritization of early surgical intervention over extensive preoperative assessment in some cases. Moreover, preoperative language assessments performed as a part of standard care were insufficient to determine the type of aphasia. Ideally, more detailed assessments, such as the Western Aphasia Battery, should be conducted preoperatively. This study included patients with grades 2, 3, and 4 gliomas. The potential for plasticity is known to differ depending on tumor malignancy grade. In grade 2 and 3 gliomas, slow-growing lesions strongly promote functional reorganization (Duffau et al., 2003, Krishna et al., 2021). In contrast, in grade 4 gliomas, although the potential for plasticity is lower, several studies have reported that reorganization can occur to some extent (Majos et al., 2017, Nakajima et al., 2020, Southwell et al., 2016). In higher-grade gliomas, however, the reorganization process may still be ongoing at the preoperative timepoint (Cargnelutti et al., 2020). Although there may be separate processes in changes in the spatial organization of functionality in lower- and higher-grade gliomas, we found that positive responses in the MFG were observed exclusively in the FLA group across grades. Further studies are needed to validate our findings and clarify differences in reorganization patterns among malignant grades.

It has been reported that the efficiency of global networks in normal brains is similar to that in brains with focal damage in several diseases, including epilepsy (Ridley et al., 2015). The recruitment of high-centrality MFG areas can be interpreted from the following point of view. The MFG areas, particularly its posterior region, are known as the functional connectivity hub of the network (Bagarinao et al., 2020, Bray et al., 2015). The brain may attempt to maintain the system-level efficiency of neural networks against tumor progression by rearranging functional areas into hub regions with rich connections to other nodes (Ridley et al., 2015). We previously found that language function could be compensated by a hub region of the network, with lesion progression to the posterior language area (Nakajima et al., 2024). Consistently, a neuroimaging study examining structural changes using longitudinal MRI after stroke suggested the importance of structural neuroplasticity in hub regions for functional recovery (Chen et al., 2021). Taken together, hub regions in the brain around the lesion might contribute to functional reorganization by supporting brain functions potentially affected by tumor progression.

## CRediT authorship contribution statement

**Riho Nakajima:** Writing – original draft, Methodology, Funding acquisition, Formal analysis, Data curation, Conceptualization. **Akitoshi Ogawa:** Writing – original draft, Formal analysis. **Masashi Kinoshita:** Writing – review & editing, Data curation. **Takahiro Osada:** Writing – original draft, Formal analysis. **Hirokazu Okita:** Writing – review & editing. **Seiki Konishi:** Writing – original draft. **Mitsutoshi Nakada:** Writing – review & editing, Supervision, Funding acquisition, Conceptualization.

## Declaration of competing interest

The authors declare that they have no known competing financial interests or personal relationships that could have appeared to influence the work reported in this paper.

## Data Availability

Data availability statement: The data that support the findings of this study are available by request from the corresponding author. The data are not publicly available due to privacy/ethical restrictions.

## Appendix A Supplementary data

Supplementary Fig. S1 Flowchart of the patient inclusion process.

Supplementary Fig. S2 Flow chart showing the number of patients included in each analysis.

Supplementary Fig. S3 Intraoperative findings of the non-FLA group excluding patients whose tumor extended to the middle frontal gyrus (MFG). (A) Patients whose tumor involved the MFG (n=16) were excluded. A map of tumor overlap is shown for the remaining patients (n=46). (B) The posterior inferior frontal gyrus (pIFG) still showed higher positive response percentages. The warm and cool colors indicate areas with high and low frequencies of positive responses, respectively. One side of a square represents 10 mm. (C) The percentage of positive responses was almost same as the non-FLA group, with 40% showing positive responses in the pIFG, and no positive responses in the MFG.

Supplementary Fig. S4 Frequency of positive responses in different malignancy grades. We divided patients into two groups, grade 2 and 3 (A) and grade 4 (B), and compared the frequencies of positive responses between the non-FLA and FLA groups. Red, positive responses; blue, normal responses. *** P < 0.001, ** P < 0.01. pIFG, posterior inferior frontal gyrus; MFG, middle frontal gyrus; FLA, frontal language area.

Supplementary Fig. S5 Locational correspondence between intraoperative findings and rsfMRI results. (A) Parcel locations of a9-46v and 8C. (B) Positive response areas in the MFG from DES in the FLA group were divided into anterior and posterior parts, corresponding to the a9-46v and 8C, respectively. rsfMRI, resting-state functional magnetic resonance imaging; MFG, middle frontal gyrus; DES, direct electrical stimulation.

Supplementary Fig. S6 Additional analyses of rsfMRI results with different thresholds. We performed three-way analysis of variance, with brain areas (8C + a9-46v vs. other regions, 8C vs. other regions, and a9-46v vs. other regions [excluding pIFG]) as a within-subject factor, and patient groups (non-FLA/FLA) and MRI scanner as between-subject factor, both treated as main effects. The analysis was repeated using different thresholds including 0.075, 0.125, and 0.150. Error bars indicate the standard error of means. **P* < 0.05, ***P* < 0.01.

Supplementary Fig. S7 Additional analyses of rsfMRI results using the right hemisphere control group. (A) Maps of tumor overlap in the age-matched control group with right cerebral hemispheric gliomas (N = 38, 46.9 ± 15.7 years). Yellow regions show the highest overlap. (B) We conducted a two-way analysis of variance with brain areas (MFG (8C + a9-46v) vs. other regions [excluding pIFG]) and MRI scanner as main effects in the left MFG (8C + a9-46v, 8C, and a9-46v). Error bars indicate the standard error of means. White triangle, 8C; Cyan triangle, a9-46v. **P* < 0.05.
