# Supplementary figures and images for: Broader functionality of language areas at the left middle frontal gyrus in patients with Broca’s area tumors
Source: Neuroimage Clin. 2025 Aug 6;48:103860. doi: 10.1016/j.nicl.2025.103860 (PMC12361790; doi:10.1016/j.nicl.2025.103860)

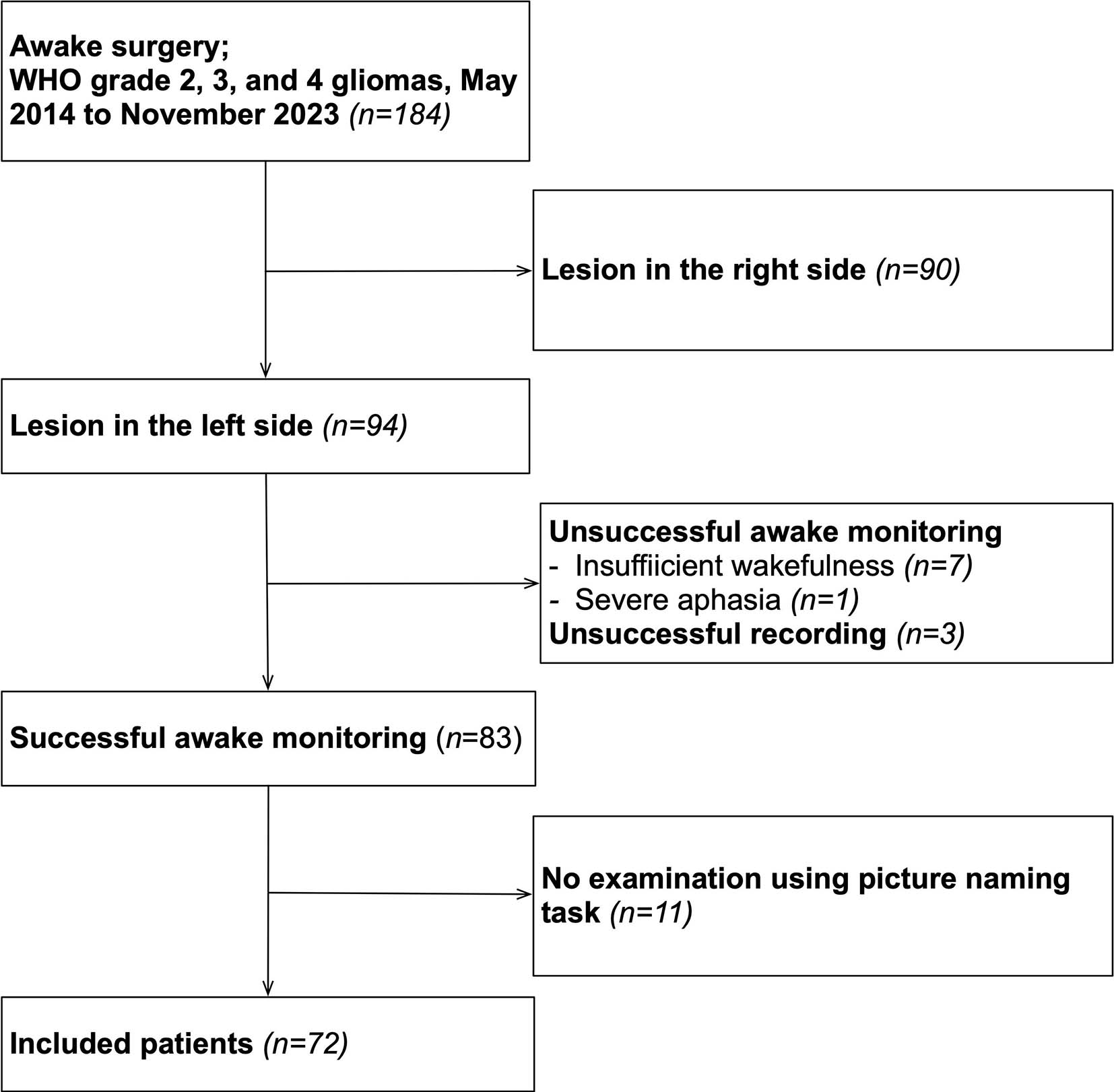

Supplement: Supplementary Fig. S1 — Flowchart of the patient inclusion process. [file mmc1.jpg]

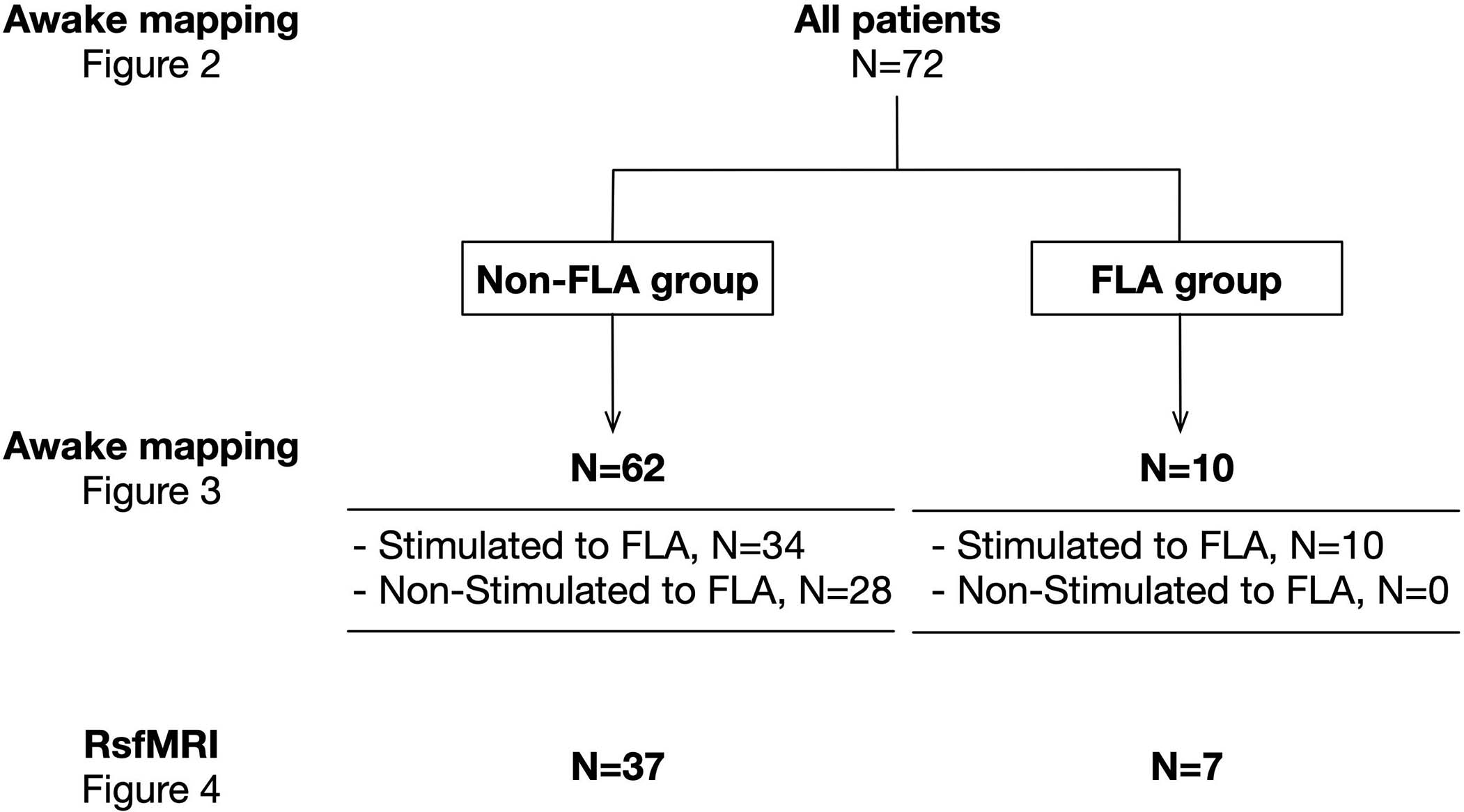

Supplement: Supplementary Fig. S2 — Flow chart showing the number of patients included in each analysis. [file mmc2.jpg]

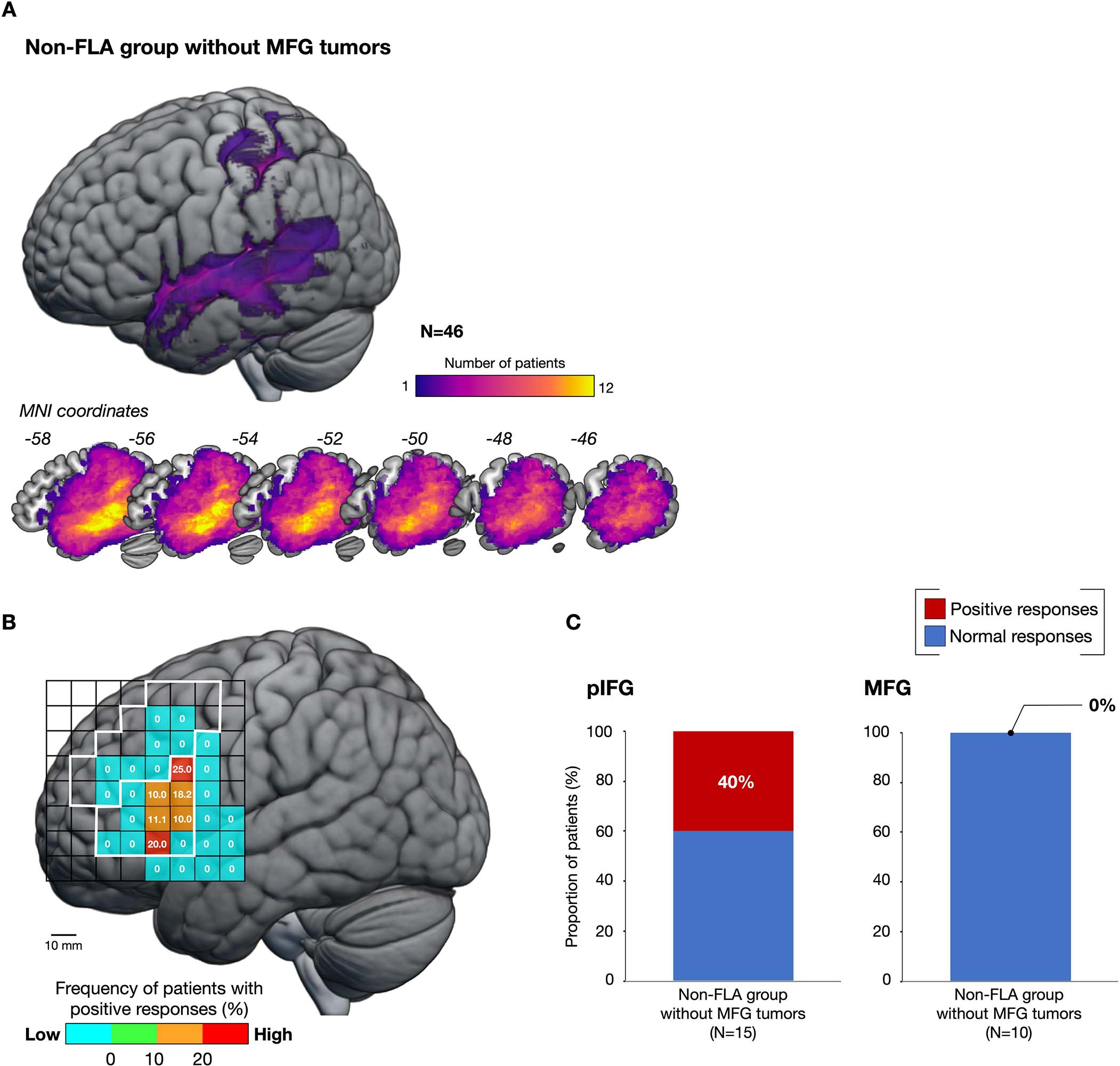

Supplement: Supplementary Fig. S3 — Intraoperative findings of the non-FLA group excluding patients whose tumor extended to the middle frontal gyrus (MFG). (A) Patients whose tumor involved the MFG (n=16) were excluded. A map of tumor overlap is shown for the remaining patients (n=46). (B) The posterior inferior frontal gyrus (pIFG) still showed higher positive response percentages. The warm and cool colors indicate areas with high and low frequencies of positive responses, respectively. One side of a square represents 10 mm. (C) The percentage of positive responses was almost same as the non-FLA group, with 40% showing positive responses in the pIFG, and no positive responses in the MFG. [file mmc3.jpg]

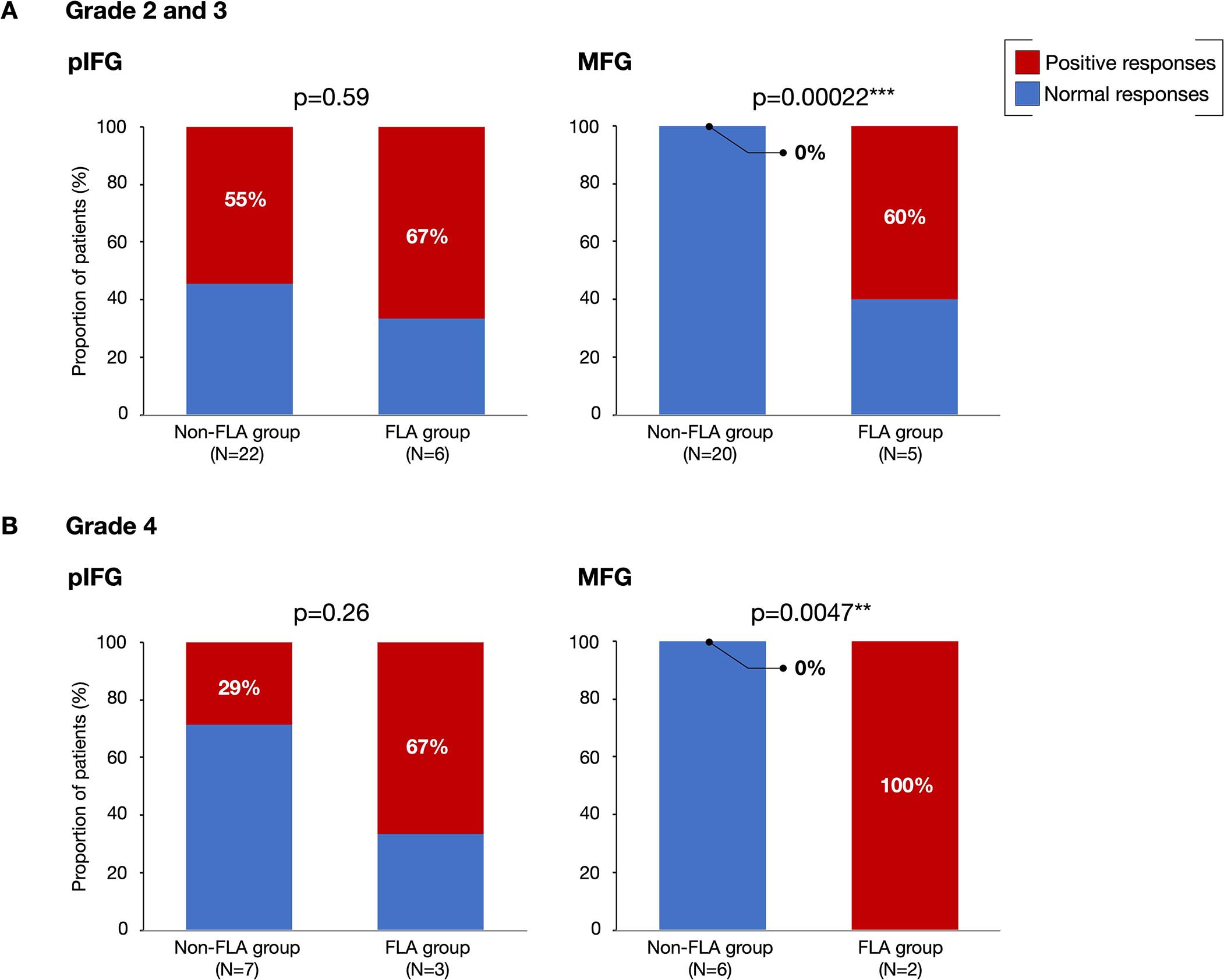

Supplement: Supplementary Fig. S4 — Frequency of positive responses in different malignancy grades. We divided patients into two groups, grade 2 and 3 (A) and grade 4 (B), and compared the frequencies of positive responses between the non-FLA and FLA groups. Red, positive responses; blue, normal responses. *** P < 0.001, ** P < 0.01. pIFG, posterior inferior frontal gyrus; MFG, middle frontal gyrus; FLA, frontal language area. [file mmc4.jpg]

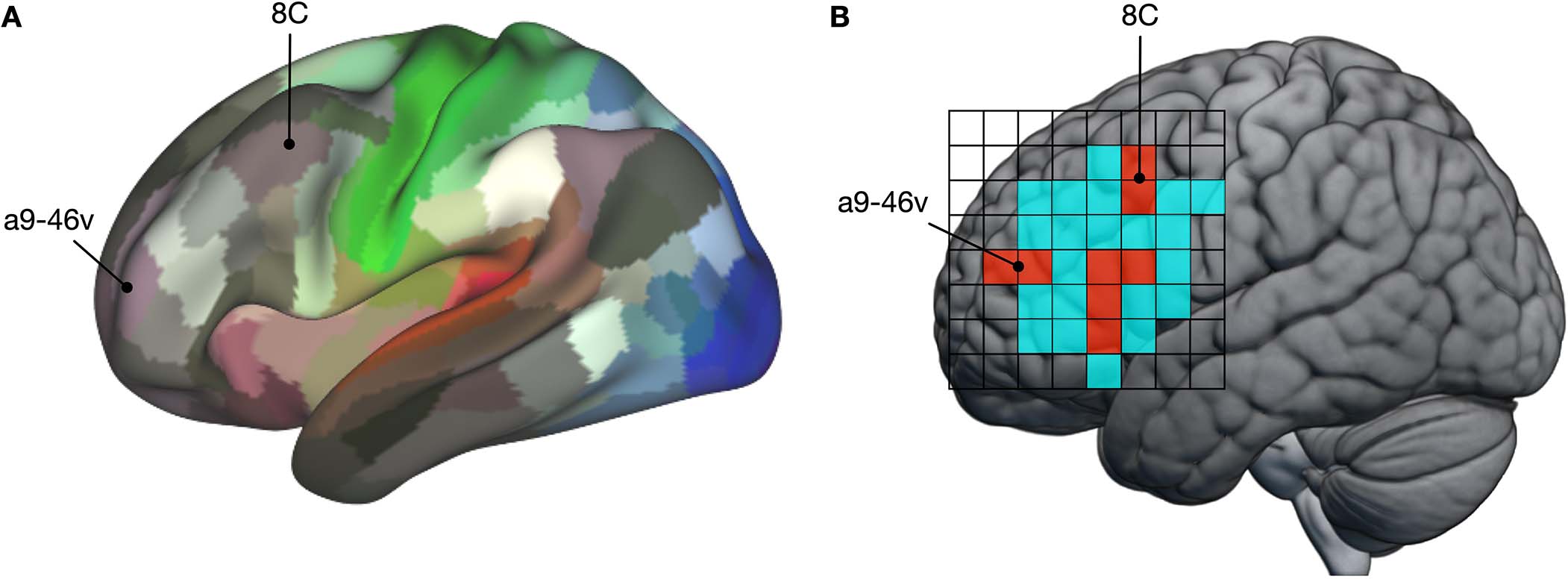

Supplement: Supplementary Fig. S5 — Locational correspondence between intraoperative findings and rsfMRI results. (A) Parcel locations of a9-46v and 8C. (B) Positive response areas in the MFG from DES in the FLA group were divided into anterior and posterior parts, corresponding to the a9-46v and 8C, respectively. rsfMRI, resting-state functional magnetic resonance imaging; MFG, middle frontal gyrus; DES, direct electrical stimulation. [file mmc5.jpg]

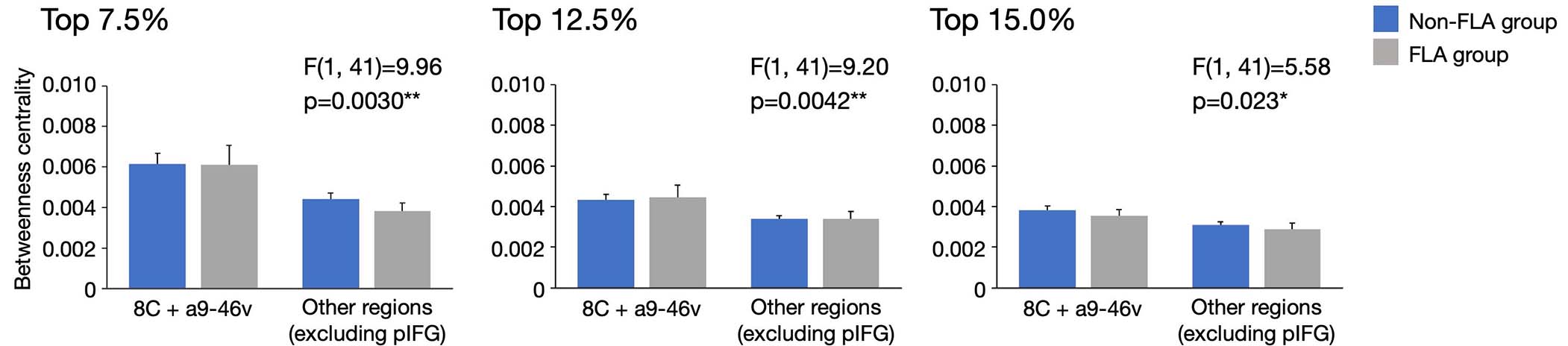

Supplement: Supplementary Fig. S6 — Additional analyses of rsfMRI results with different thresholds. We performed three-way analysis of variance, with brain areas (8C + a9-46v vs. other regions, 8C vs. other regions, and a9-46v vs. other regions [excluding pIFG]) as a within-subject factor, and patient groups (non-FLA/FLA) and MRI scanner as between-subject factor, both treated as main effects. The analysis was repeated using different thresholds including 0.075, 0.125, and 0.150. Error bars indicate the standard error of means. *P < 0.05, **P < 0.01. [file mmc6.jpg]

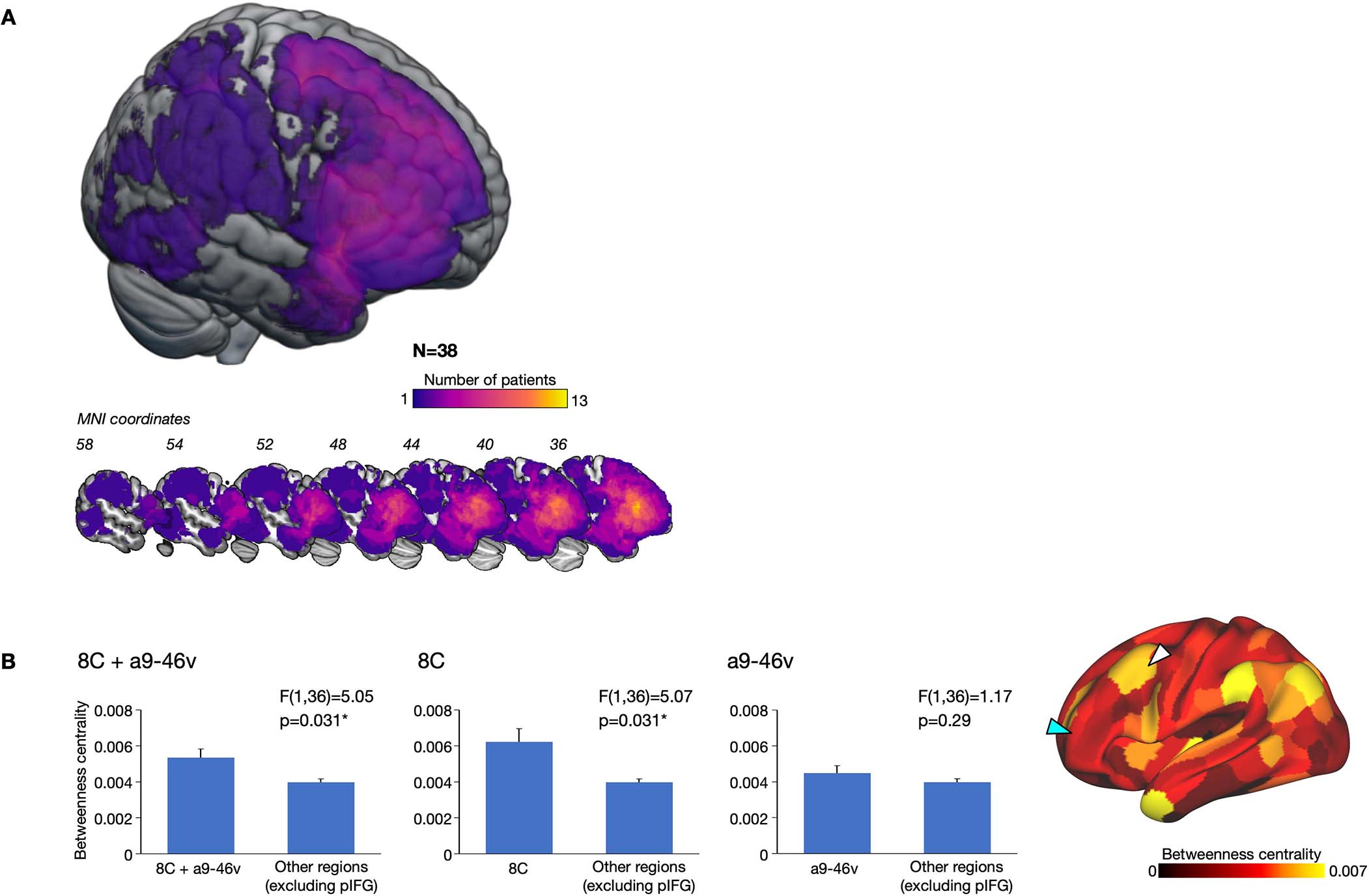

Supplement: Supplementary Fig. S7 — Additional analyses of rsfMRI results using the right hemisphere control group. (A) Maps of tumor overlap in the age-matched control group with right cerebral hemispheric gliomas (N = 38, 46.9 ± 15.7 years). Yellow regions show the highest overlap. (B) We conducted a two-way analysis of variance with brain areas (MFG (8C + a9-46v) vs. other regions [excluding pIFG]) and MRI scanner as main effects in the left MFG (8C + a9-46v, 8C, and a9-46v). Error bars indicate the standard error of means. White triangle, 8C; Cyan triangle, a9-46v. *P < 0.05. [file mmc7.jpg]
