# Supplementary material for: Broader functionality of language areas at the left middle frontal gyrus in patients with Broca’s area tumors
Source: Neuroimage Clin. 2025 Aug 6;48:103860. doi: 10.1016/j.nicl.2025.103860 (PMC12361790; doi:10.1016/j.nicl.2025.103860)
Supplement: Supplementary Table [file mmc8.docx]

**Supplementary Table 1**. MNI coordinates for center of positive points

| No | Responses | MNI coordinates | | |
| --- | --- | --- | --- | --- |
|  |  | X | Y | Z |
| 1 | Anomia | -54.5 | 24.5 | 33 |
| 2 | Anomia | -61.5 | 18 | 14 |
| 3 | Anomia | -61 | 15.5 | 14.5 |
| 4 | Anomia | -61.5 | 17.5 | 14 |
| 5 | Anomia | -61.5 | 18 | 13.5 |
| 6 | Anomia | -59.5 | 26 | 5 |
| 7 | Anomia | -61.5 | 17.5 | 13.5 |
| 8 | Anomia | -61 | 17.5 | 9.5 |
| 9 | Anomia | -59.5 | 24 | 8 |
| 10 | Anomia | -61 | 20 | 18 |
| 11 | Anomia | -58 | 28.5 | 19.5 |
| 12 | Anomia | -61 | 17 | 24 |
| 13 | Anomia | -60 | 25 | 11 |
| 14 | Anomia | -62 | 17.5 | 24 |
| 15 | Anomia | -42 | 14 | 54 |
| 16 | Anomia | -57 | 28 | 19.5 |
| 17 | Anomia | -56 | 23 | -1 |
| 18 | Anomia | -53 | 15 | 43 |
| 19 | Phonemic | -56 | 27 | 0 |
| 20 | Semantic | -62 | 15.5 | 14.5 |
| 21 | Semantic | -57.5 | 30 | 13 |
| 22 | Semantic | -59 | 26 | 8 |
| 23 | Semantic | -59.5 | 16.5 | 5 |
| 24 | Semantic | -61 | 23.5 | 20.5 |
| 25 | Semantic | -59.5 | 24 | 3.5 |
| 26 | Semantic | -58 | 26.5 | 3.5 |
| 27 | Semantic | -49 | 43 | 16 |
| 28 | Semantic | -47 | 51 | 18 |
| 29 | Semantic | -59.5 | 23 | 8 |
| 30 | Semantic | -59.5 | 21.5 | 2 |
| 31 | Semantic | -54 | 17.5 | 42 |
| 32 | Speech arrest | -54.5 | -3 | 49.5 |
| 33 | Speech arrest | -63.5 | 3.5 | 28 |
| 34 | Speech arrest | -63.5 | 4.5 | 19 |
| 35 | Speech arrest | -63.5 | 4.5 | 13 |
| 36 | Speech arrest | -56 | 42 | -8 |
| 37 | Speech arrest | -25 | -2 | 71 |
| 38 | Speech arrest | -45 | 4 | 53.5 |
| 39 | Anarthria | -56 | -2 | 38 |
| 40 | Anarthria | -63 | 2 | 20 |
| 41 | Anarthria | -56 | -3 | 48 |
| 42 | Anarthria | -57 | -3 | 48 |
| 43 | Anarthria | -61 | 3 | 36 |
| 44 | Anarthria | -64 | 4 | 19 |
| 45 | Anarthria | -62 | 5 | 24 |
| 46 | Anarthria | -65 | -11 | 24 |
| 47 | Anarthria | -65 | -11 | 24 |
| 48 | Anarthria | -64 | 4 | 16 |
| 49 | Anarthria | -65 | 2 | 16 |
| 50 | Anarthria | -53 | -6 | 51 |
| 51 | Anarthria | -64 | 6 | 14 |
| 52 | Anarthria | -64 | 6 | 14 |
| 53 | Anarthria | -63 | 5 | 30 |
| 54 | Anarthria | -62 | 5 | 30 |
| 55 | Anarthria | -62 | 5 | 13 |
| 56 | Anarthria | -65 | -7 | 25 |
| 57 | Anarthria | -67 | -10 | 20 |
| 58 | Anarthria | -66 | -10 | 20 |
| 59 | Anarthria | -61 | -13 | 42 |
| 60 | Anarthria | -64 | 3 | 24 |
| 61 | Anarthria | -64 | 3 | 14 |
| 62 | Anarthria | -61 | 3 | 35 |
| 63 | Anarthria | -62 | 7 | 24 |
| 64 | Anarthria | -62 | 5 | 24 |
| 65 | Anarthria | -47 | -7 | 57 |
| 66 | Anarthria | -61 | 3 | 36 |
| 67 | Anarthria | -57 | -3 | 46 |
| 68 | Anarthria | -65 | -11 | 24 |
| 69 | Anarthria | -59 | 4 | 41 |
| 70 | Anarthria | -64 | 3 | 14 |
| 71 | Anarthria | -64 | 3 | 14 |
| 72 | Anarthria | -63 | 5 | 24 |
| 73 | Anarthria | -64 | 3 | 18 |
| 74 | Anarthria | -63 | 5 | 17 |
| 75 | Anarthria | -64 | 3 | 20 |
| 76 | Anarthria | -64 | 5 | 33 |
| 77 | Anarthria | -64 | 5 | 29 |
| 78 | Anarthria | -56 | 1 | 47 |
| 79 | Anarthria | -62 | -3 | 38 |
| 80 | Anarthria | -62 | 5 | 30 |
| 81 | Anarthria | -62 | 4 | 21 |
| 82 | Anarthria | -63 | 4 | 13 |
| 83 | Anarthria | -55 | -6 | 49 |
| 84 | Anarthria | -61 | 5 | 22 |
| 85 | Anarthria | -59 | -1 | 41 |
| 86 | Anarthria | -59 | 1 | 40 |
| 87 | Anarthria | -64 | 5 | 14 |
| 88 | Anarthria | -64 | 4 | 20 |
| 89 | Anarthria | -64 | 3 | 14 |
| 90 | Anarthria | -64 | 3 | 28 |
| 91 | Anarthria | -58 | 3 | 46 |
| 92 | Anarthria | -52 | 3 | 50 |
| 93 | Anarthria | -62 | 7 | 24 |
| 94 | Anarthria | -64 | 6 | 30 |
| 95 | Anarthria | -62 | 6 | 24 |
| 96 | Anarthria | -62 | 7 | 34 |
